# Long Non-Coding RNAs in Malignant Human Brain Tumors: Driving Forces Behind Progression and Therapy

**DOI:** 10.3390/ijms26020694

**Published:** 2025-01-15

**Authors:** Dakun Pei, Dandan Zhang, Yan Guo, Hongbo Chang, Hongjuan Cui

**Affiliations:** State Key Laboratory of Resource Insects, Medical Research Institute, Southwest University, Chongqing 400715, China; kun2139@email.swu.edu.cn (D.P.); zhangdandan1234@email.swu.edu.cn (D.Z.); gy980423@email.swu.edu.cn (Y.G.); a1103668795@email.swu.edu.cn (H.C.)

**Keywords:** long non-coding RNA, glioblastoma, medulloblastoma, meningioma, radiosensitivity, chemosensitivity

## Abstract

Long non-coding RNAs (lncRNAs) play a pivotal role in regulating gene expression and are critically involved in the progression of malignant brain tumors, including glioblastoma, medulloblastoma, and meningioma. These lncRNAs interact with microRNAs (miRNAs), proteins, and DNA, influencing key processes such as cell proliferation, migration, and invasion. This review highlights the multifaceted impact of lncRNA dysregulation on tumor progression and underscores their potential as therapeutic targets to enhance the efficacy of chemotherapy, radiotherapy, and immunotherapy. The insights provided offer new directions for advancing basic research and clinical applications in malignant brain tumors.

## 1. Introduction

Non-coding RNAs (ncRNAs) represent a diverse class of transcripts comprising more than 90% of the RNA content within the human genome. Since their discovery, ncRNAs have been recognized as critical regulators of numerous biological processes across various cell types and tissues. Aberrant expression of ncRNAs has been associated with cancer and a multitude of other diseases, underscoring their potential utility as biomarkers and therapeutic targets. While extensive research has centered on the role of microRNAs (miRNAs) in human cancers, the considerable potential of other types of non-coding RNAs, such as long non-coding RNAs (lncRNAs) and miRNAs, ought not to be disregarded [1,2,3,4].

In cancer, lncRNAs play crucial roles in regulating tumor progression, exhibiting diverse functions that enable them to contribute to both oncogenic and tumor-suppressive processes. These effects are often achieved by regulating cell proliferation, migration, invasion, and epigenetic modifications [5,6]. In addition, multiple lncRNAs regulate tumor initiation and metastasis by modulating cancer stem cells’ (CSCs) stemness and the epithelial-to-mesenchymal transition (EMT) process. The EMT enhances the migratory and invasive properties of cancer cells. CSCs, which act as reservoirs for tumor initiation, activate the EMT program, generating cancerous mesenchymal cells with invasive and metastatic potential [7].

Contrary to traditional perspectives, certain lncRNAs can “encode” micropeptides, which participate in regulating gene transcription and signal transduction. These micropeptides hold substantial promise as diagnostic biomarkers, prognostic indicators, and therapeutic targets [8]. Moreover, beyond their direct roles as tumor regulators in various cancers, lncRNAs can interact with miRNAs through competitive binding and influence miRNA biogenesis, forming crosstalk within miRNA networks. This interaction further broadens the functional scope of lncRNA [9,10,11]. Given the strong correlation between lncRNA dysregulation and cancer progression, lncRNAs have emerged as valuable molecular biomarkers for diagnosis, prognosis, and potential therapeutic targets [12,13,14,15]. Additionally, lncRNAs play pivotal roles in immunotherapy and in modulating the sensitivity of tumors to chemotherapy and radiotherapy [16,17,18,19].

Malignant brain tumors present significant health challenges due to their low survival rates and limited therapeutic options, particularly glioblastoma (GB), medulloblastoma (MB), and meningioma, which affect both adults and children [20]. GB, known as the most common primary malignant brain tumor, generally has a median survival time of less than two years. Furthermore, many new therapeutic agents have failed to demonstrate efficacy in late-stage clinical trials [20,21]. MB accounts for nearly 20% of all pediatric brain tumors, with a median onset age of seven years. It often recurs as a drug-resistant disease and is associated with high morbidity and mortality. Meningioma is the most common primary intracranial tumor in adults, with its incidence rising due to an aging population. While most meningiomas exhibit benign behavior, a subset shows biological aggressiveness and treatment resistance, leading to significant neurological morbidity and even mortality [22].

Given the regulatory functions of lncRNAs and their potential as therapeutic targets, their application in malignant brain tumors should not be underestimated. This review summarizes recent advancements in lncRNA research over the past five years, focusing on their regulatory roles, impact on drug resistance, diagnostic and prognostic utility, and therapeutic strategies in malignant brain tumors, particularly GB, MB, and malignant part of meningiomas. The aim is to provide a concise reference to facilitate future studies and clinical applications.

## 2. Research Progress on the Function of lncRNA in Malignant Brain Tumors

### 2.1. The Biological Functions of lncRNA in Malignant Brain Tumors

**Figure 1:**
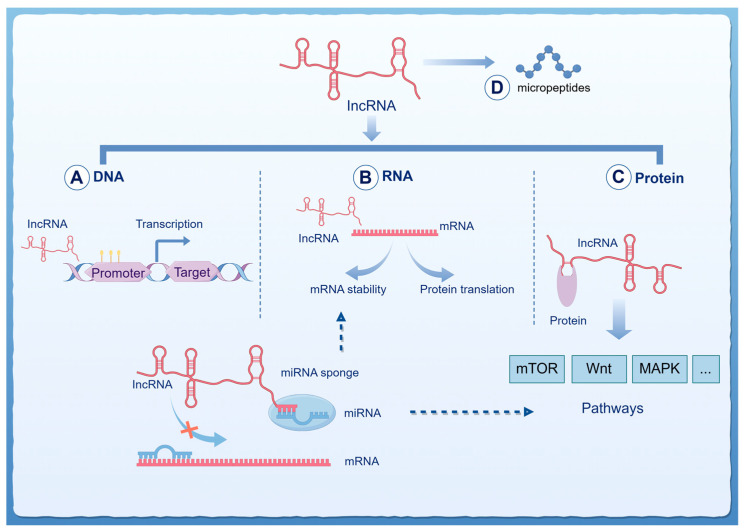
The biological functions of lncRNA in malignant brain tumors. (**A**) LncRNAs regulate the expression of target genes by interacting with DNA and forming complexes. (**B**) They can also affect mRNA stability and gene transcription by binding to mRNA. (**C**) LncRNAs have the ability to interact with proteins, influencing signaling pathways (such as mTOR, Wnt, MAPK, etc.). Notably, lncRNAs can act as competitive endogenous RNAs (ceRNAs), sequestering miRNAs (acting as “sponges”) to prevent miRNA binding to their target mRNAs, thereby influencing mRNA stability and indirectly regulating signaling pathways. (**D**) LncRNAs possess “coding” functions, forming micropeptides with potential biological activities.

To date, a substantial number of lncRNAs have been identified. Version 6.0 of the NONCODE database includes annotations for 173,112 human lncRNAs [23]. Beyond their abundance, the diversity of lncRNAs is also reflected in their functional roles and the multiple ways in which they regulate gene expression [24] (Figure 1). In malignant brain tumors, lncRNAs can directly bind to DNA or regulate chromatin as components of DNA-binding protein complexes [5]. For example, in mouse cerebellar granule cells (CB) and human medulloblastoma cell line (DAOY), Snhg15 forms RNA/DNA triplex structures and interacts with DNMT1, recruiting it to the Ncam1 promoter, which facilitates targeted DNA methylation and potentially regulates cellular motility [25]. LncRNAs can also interact with mRNA, influencing translation and RNA stability, thus regulating cancer progression. In medulloblastoma, HHIP-AS1 directly binds to the mRNA of Dynein Cytoplasmic 1 Light Intermediate Chain 2 (DYNC1I2) and attenuates its degradation by hsa-miR-425-5p to promote mitosis and facilitate tumor [26].

Furthermore, lncRNAs regulate cancer progression through interactions with miRNAs. In brain cancers, for instance, lncRNAs function as competitive endogenous RNAs (ceRNAs) and molecular sponges for miRNAs, thereby indirectly modulating multiple molecular signaling pathways, including Wnt/β-catenin, PI3K/AKT/mTOR, MAPK kinase, and NF-κB pathways [27]. By interacting with proteins, lncRNAs serve as pathway regulators or scaffolds to modulate brain tumor progression [28]. For example, LINC00998 binds to CBX3 in glioblastoma, inhibiting GB cell proliferation through the c-Met/AKT/mTOR axis and improving patient survival rates [29]. Similarly, MDHDH acts as a scaffold for MDH2 and PSMA1, regulating NAD^+^ metabolism and autophagy to suppress tumor progression [30].

LncRNAs can also “encode” micropeptides that play a role in malignant brain tumors [31,32]. In medulloblastoma, for instance, LINC00888 encodes a microprotein that is significantly upregulated in MYC-driven medulloblastoma samples and facilitates the survival of MB cells [33]. In GB, the AF127577.4 encodes an endogenous micropeptide that modulates the interaction between ERK2 and METTL3, reducing GB cell proliferation and correlating with clinical tumor grading [34].

Through their diverse molecular functions, lncRNAs play a pivotal role in regulating key processes in malignant brain tumors, including cell proliferation, anti-apoptosis, immune evasion, angiogenesis, migration, metabolic dysregulation, and epigenetic regulation.

### 2.2. LncRNAs Regulate the Progression of Malignant Brain Tumors and Relate to Prognosis

LncRNAs, through their diverse molecular functions, are involved in nearly all cellular processes and participate in numerous signaling pathways, playing key roles in the initiation and progression of various cancers [35]. The strong correlation between lncRNA dysregulation and tumor progression underscores their potential as both biomarkers and therapeutic targets [36]. This is particularly true in malignant brain tumors, where the diverse functions of lncRNAs are critical in regulating tumor stemness, growth, proliferation, migration, invasion, and the epithelial-to-mesenchymal transition (EMT). These processes are closely linked to patient prognosis and are further amplified through crosstalk with miRNA networks, thereby expanding the range of potential molecular biomarkers [37,38].

#### 2.2.1. Tumor Stemness

Tumor stem cells, serving as reservoirs for cancer, play a pivotal role in tumor initiation through self-renewal and stemness regulation, often contributing to therapeutic resistance. LOXL1-AS1 and Miat regulate cancer stemness and tumor initiation processes in medulloblastoma [39,40]. Lowering linc-RoR expression curtails cell growth, boosts sensitivity to DNA damage, and decreases cancer stem cells’ (CSCs) marker levels. In contrast, its overexpression promotes tumor cell proliferation and increases the proportion of CSCs [41]. The expression of LINC00461 is markedly elevated in stem cell-like GB cells, correlating with cell proliferation and drug resistance [42]. Similarly, the expression of INHEG is elevated in glioblastoma stem cells (GSCs), aiding in self-renewal and tumorigenic processes by modulating rRNA 2’-O-methylation [43].

Hypoxia is linked to poor prognosis in many cancers. GSCs are often located in hypoxic regions, where depletion of LUCAT1 impairs GSC self-renewal and promotes glioblastoma progression by enhancing hypoxia-inducible factor-1α (HIF-1α) activity [44]. Hypoxia-induced MIR210HG predicts poor prognosis and regulates hypoxia-mediated glioblastoma stemness, temozolomide (TMZ) resistance, and invasion [45]. HULC stabilizes the FOXM1 protein through ubiquitination and upregulates AGR2 and HIF-1α expression, promoting glycolysis, maintaining GSC stemness, and enhancing GSC tumorigenicity. These findings suggest that HULC is a potential therapeutic target in glioblastoma [46].

#### 2.2.2. Cell Proliferation and Metastasis

**Figure 2:**
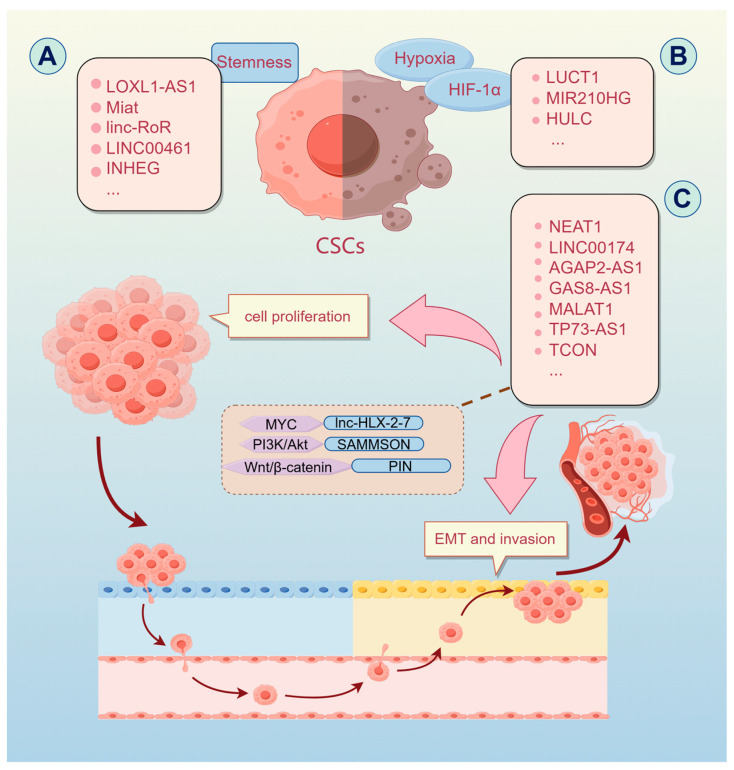
lncRNAs regulate the progression of malignant brain tumors. LncRNAs participate in the progression of malignant brain tumors through various mechanisms, including the maintenance of cancer stemness, rapid cell proliferation, migration, and invasion. (**A**) In cancer stem cells (CSCs), LOXL1-AS1, Miat, linc-RoR, and others regulate tumor stemness and self-renewal. (**B**) Under hypoxic conditions, LUCAT1, MIR210HG, and HULC promote the maintenance of stem cell properties and tumor formation by activating the HIF-1α pathway. (**C**) Various lncRNAs, including NEAT1, LINC00174, and AGAP2-AS1, are widely involved in the regulation of cell proliferation and migration. For example, lnc-HLX-2-7 enhances medulloblastoma proliferation by forming a positive feedback loop with MYC. SAMMSON blocks EMT and tumor progression by inhibiting the PI3K/Akt signaling pathway, and MIR31HG promotes glioblastoma proliferation by activating the Wnt/β-catenin signaling pathway.

One of the characteristics of malignant tumors is a fast cell proliferation rate (Figure 2). In recent years, many studies have been on regulating brain tumor cell proliferation by lncRNAs. In medulloblastoma, depletion of lnc-HLX-2-7 significantly reduces cell proliferation and tumorigenicity while inducing apoptosis [47]. The same researchers demonstrated that lnc-HLX-2-7 functions as an enhancer to upregulate HLX expression and mediates the formation of a positive feedback loop with MYC, thereby promoting tumor progression [48]. NEAT1, a key regulatory factor, significantly suppresses GB proliferation and glycolysis when knocked down, and its expression is inversely correlated with overall survival in GB patients [49].

Additionally, LINC00174 regulates GB cell proliferation and serves as an independent prognostic marker for glioblastoma patients, with significant correlations to tumor grading and patient survival rates [50].

Metastasis and spread of tumors are achieved through migration and invasion, and lncRNA exerts a significant role in this process [51]. AGAP2-AS1, a carcinogenic lncRNA, regulates GB cell proliferation, apoptosis, migration, and invasion. It has been identified as both a prognostic biomarker and a potential therapeutic target for GB patients [52]. By binding to the transcription factor NF-κb1 and activating IL4R, LBX2-AS1 facilitates glioblastoma metastasis and angiogenesis, suggesting a potential target for GB therapy [53].

The epithelial-to-mesenchymal transition (EMT) mechanism acts as a key driver of tumor cell metastasis, with dysregulated EMT processes closely associated with tumor migration and invasion [54]. In GB cells, GAS8-AS1 downregulates NEAT1, inhibiting cell proliferation and invasion [55]. Through the promotion of ZEB1 expression, MALAT1 induces EMT, thereby contributing to the resistance of GB cells to temozolomide (TMZ) [56]. The downregulation of TP73-AS1 in medulloblastoma results in apoptosis and curtails cell proliferation and migration, proposing that TP73-AS1 could act as a prognostic marker and therapeutic target [57]. Depletion of SAMMSON inhibits the PI3K/Akt pathway, suppressing EMT and tumor progression [58].

Conversely, PIN is downregulated in GB tissues and cells and inhibits proliferation, invasion, and EMT by blocking the Wnt/β-catenin signaling pathway [59]. Bioinformatic analyses and experimental validation have identified a novel lncRNA, TCONS_00020456 (TCON), as a prognostic and diagnostic biomarker. TCON functions as a tumor suppressor by inhibiting cell proliferation, migration, and invasion, and it can suppress the EMT process in vivo [60].

In brain tumors, lncRNAs modulate tumor progression by regulating epigenetic processes such as methylation and ubiquitination. Despite being primarily benign in nature, meningiomas with benign histological findings can occasionally present with aggressive biological behavior, hypomethylation of the FOXC1 promoter, and its upstream lncRNA transcript FOXCUT is associated with tumor progression [61]. In glioblastoma, the N6-methyladenosine (m6A)-modified LINREP interacts with the PTBP1/HuR complex and protects PTBP1 from ubiquitin-mediated degradation, thereby promoting tumor progression and potentially serving as a novel therapeutic target [62]. The research discovered a new cancer-related lncRNA called EPAT that prevents USP16 from deubiquitinating H2A and suppresses the expression of certain genes. Removing EPAT boosts USP16’s ability to halt the cell cycle and promote cellular aging, thereby reducing tumor growth. Its expression level is positively correlated with tumor grade and predicts poor prognosis for patients [63]. RMST enhances the SUMOylation of FUS, regulates mitochondrial autophagy, and promotes tumor progression, making it a promising prognostic factor for glioma patients [64].

In addition to these roles, lncRNAs play a critical regulatory role in predicting patient survival through liquid biopsy [65]. In ependymoma (EPN), TRERNA1 is highly expressed, particularly in pediatric posterior fossa EPN, and is associated with shorter progression-free survival [66]. Using non-invasive liquid biopsy, blood samples from 35 newly diagnosed GB patients and 15 healthy individuals were analyzed, revealing that the expression of lncRNA565 and lncRNA641 could serve as prognostic biomarkers for GB patients [67]. Furthermore, DLEU1, an oncogenic lncRNA, inhibits ferroptosis in glioblastoma [68].

#### 2.2.3. Multiple Feature-Related lncRNA Prognostic Models from Database Mining

By conducting multi-dimensional analysis of lncRNAs from clinical samples and various databases, cost-effective and efficient models can be developed to screen molecular biomarkers and construct related risk models. Over the past five years, several studies have highlighted lncRNAs as potential prognostic markers associated with various characteristics, including immunity, epigenetic modifications, and metabolism.

Immune-related long non-coding RNAs (ir-lncRNAs) are crucial in tumor progression and prognosis assessment. Multidimensional database analyses have led to the development of prognostic models incorporating several ir-lncRNAs, which assess subtype-specific prognostic value for glioblastoma, clinical features, immune infiltration pathways, and chemotherapy efficacy. These models demonstrate promising clinical value in predicting GB prognosis and immune landscapes [69,70,71,72,73].

Methylation modifications, a frontier topic in epigenetics, continue to receive considerable research attention. Methylation is significantly associated with cancer patient prognosis. Machine learning and multi-database analyses have revealed that methylation-related lncRNA cohorts and risk models have significant potential in predicting the prognosis of glioma patients [74,75]. N6-methyladenosine, the most prevalent methylation modification in lncRNAs, has been implicated in various clinical contexts. Several studies have identified m6A-related lncRNAs as potential biomarkers for predicting overall survival in glioma patients [76,77,78]. The m6A-mediated WEE2-AS1 promotes glioblastoma progression by stabilizing RPN2, positioning it as a potential prognostic biomarker and therapeutic target for GB [79]. A genomic analysis of pediatric medulloblastoma identified N6-methyladenosine-dependent lncRNA features linked to molecular subtypes, immune cell infiltration, and prognosis [80].

Metabolic reprogramming is a hallmark of cancer, and identifying lncRNA signatures related to glycolysis and amino acid metabolism has shown a significant influence on predicting poor prognosis and shorter overall survival in glioma patients, thus serving as potential future therapeutic targets [81,82]. The dynamic interplay between sphingolipid metabolism and tumor immune phenotypes offers potential clinical applications, with the establishment of prognostic models that include sphingolipid metabolism-related lncRNAs, providing multi-faceted utility for diagnostic and prognostic purposes in glioma patients [83].

Pyroptosis, a regulated form of cell death characterized by iron-dependent lipid peroxidation in membrane phospholipids, has been widely studied for its involvement in cancer and neurodegenerative diseases. Pyroptosis may be effective for tumors that escape immunotherapy and resist chemotherapy. An analysis of three independent cohorts based on TCGA and CGGA databases used an algorithm to identify pyroptosis-related lncrnas (PRLS), and the correlation between chemotherapy and immunotherapy was discussed. The prognostic module showed stable and accurate performance in predicting 1-, 3-, and 5-year outcomes. Patients with a high PRLS score indicate a more abundant immune infiltration, higher expression of immune checkpoint genes, and a better response to immunotherapy but a worse response to chemotherapy. These features provide new perspectives for the clinical management and precision therapy of GB [84]. Analysis of the database in other studies also showed that pyroptosis-related lncRNAs may be a dependent prognostic factor, which may be related to the immune response of glioma, help to evaluate the prognosis and treatment mode of patients, and can be further applied in clinical practice [85,86].

For recurrent tumors, database analysis and single-cell sequencing data mining identified OBI1-AS1 as an astrocyte marker that may play a role in glioma recurrence and progression [87]. Using the GB PDX model, researchers simulated the long-term development of recurrent tumors under continuous radiation therapy, discovering lncRNAs connected to acquired radiation resistance in recurrent glioblastoma. This facilitates upcoming preclinical therapy testing to combat treatment resistance in GB patients [88]. Although the vast majority of meningiomas are not malignant, identifying biomarkers that can predict their recurrence still holds clinical value, and lnc-GOLGA6A-1, ISLR2, and AMH demonstrated high prognostic potential for predicting meningioma recurrence, with lnc-GOLGA6A-1 being the most prominent [89].

Other studies on prognostic lncRNA features in models include pyroptosis, EMT [90,91,92,93], ferroptosis [94,95,96], transcription factors (TFs) [97], tumor necrosis factor (TNF) [98], aging [99], angiogenesis [100], oxidative stress [101], copper-induced cell death [102], and apoptosis [103]. The integration of diverse datasets in these models facilitates further investigation into the regulation of lncRNAs, helps assess immune infiltration, and evaluates the efficacy of various systemic anti-tumor treatments.

### 2.3. lncRNA-miRNA Regulatory Network in Malignant Brain Tumors

Notably, one of the most prominent hypotheses for understanding the generalized mechanisms of lncRNA functionality is the competitive endogenous RNA (ceRNA) theory. Recent studies have highlighted the crosstalk between miRNAs and other non-coding RNAs with lncRNAs, forming ceRNA networks. These ceRNAs share microRNA response elements (MREs), exerting multiple regulatory effects. LncRNA can bind to specific miRNA binding sites, thereby regulating miRNA expression and function. This mechanism has been extensively studied in various cancers, including breast cancer, melanoma, colorectal cancer, and non-small-cell lung cancer [104,105,106,107,108,109].

In malignant brain tumors, numerous studies have shown that lncRNAs can exert indirect regulatory effects on tumor initiation and progression through ceRNA networks by modulating miRNA expression (Table 1).

We observed that the same lncRNA can regulate different miRNAs, while different lncRNAs can regulate the same miRNA through distinct mechanisms. Furthermore, the same lncRNA-miRNA interactions can influence multiple signaling pathways. This complex and extensive regulatory network significantly broadens the potential applications and mechanisms of lncRNAs.

In conclusion, the diverse functions of lncRNAs play a pivotal role in regulating brain tumors, particularly through ceRNA network crosstalk, which amplifies and extends their regulatory impact. This approach provides broader perspectives for exploring lncRNA functionality and enhances their potential as molecular biomarkers and therapeutic targets.

## 3. Research Progress of lncRNAs in the Treatment of Malignant Brain Tumors

### 3.1. Chemosensitivity

Brain tumors are highly malignant and pose a significant threat to health, often resulting in substantial increases in global mortality and morbidity. The development of drug resistance further complicates the treatment of brain tumors. Non-coding RNAs are widely involved in regulating drug resistance, particularly in the case of temozolomide, a key chemotherapeutic agent used for glioblastoma [181]. In medulloblastoma, knockout of the NEAT1 increases chemosensitivity and enhances cisplatin-induced apoptosis through miR-23a-3p [182]. Etoposide, a cell cycle-specific anticancer drug, acts by inhibiting DNA topoisomerase II. IGF2BP2 promotes the ubiquitination of FOXO1, leading to resistance of GB cells to etoposide [183]. Inhibition of the long non-coding RNA CRNDE increases the chemosensitivity of medulloblastoma cells by targeting miR-29c-3p [112].

Temozolomide (TMZ), as a first-line therapeutic agent for glioblastoma (GB), is worth discussing in depth. Although TMZ demonstrates significant therapeutic potential in the early stages of treatment, many GB patients gradually develop resistance to TMZ, leading to disease progression and death [184].

#### 3.1.1. TMZ Resistance

LncRNA plays a role in TMZ resistance; HOTAIR is studied in the dysregulation of several cellular functions, such as apoptosis, cell cycle, epithelial–mesenchymal transition (EMT), autophagy, self-renewal, and metabolism, all of which are associated with chemotherapy resistance [185]. To address TMZ resistance caused by the HOTAIR/EZH2 pathway, Yang et al. developed the small-molecule drug EPIC-0628, which enhances TMZ efficacy [186]. Zhao et al. designed EPIC-0412, which physically disrupts the interaction between HOTAIR and EZH2, restoring TMZ sensitivity in GB in vivo models [187]. Similarly, Xin et al. designed a novel compound, EPIC-0307, which reestablishes TMZ sensitivity by targeting the PRADX-EZH2 axis [188]. Furthermore, GB with high HOTAIR expression shows increased sensitivity to methotrexate, suggesting that methotrexate could serve as a potential therapeutic agent for patients with elevated HOTAIR expression levels [189].

One of the factors complicating the signaling networks involved in GB progression and TMZ resistance is the presence of feedback loops. The RMRP/ZNRF3 axis and the Wnt/β-catenin signaling pathway form a positive feedback loop that regulates TMZ resistance in glioma [190]. The HOXD-AS2-STAT3 positive feedback loop plays a key role in regulating TMZ sensitivity. Lower levels of HOXD-AS2 make glioblastoma more sensitive to temozolomide; STAT3-induced HOXD-AS2 interacts with the IGF2BP2 protein, forming a complex that subsequently upregulates STAT3 signaling, thereby creating a positive feedback loop that regulates TMZ sensitivity in glioblastoma [191].

Other lncRNAs also play significant roles in regulating TMZ resistance in GB. For example, PDIA3P1 has been shown to reduce GB cell sensitivity to TMZ treatment. The small-molecule NEF blocks the upregulation of PDIA3P1 and enhances the synergistic effect when combined with specific concentrations of TMZ [192]. LINC01956’s dynamic structural remodeling enhances MGMT methylation, promoting TMZ resistance in glioblastoma [193]. Additionally, SOX2OT and ADAMTS9-AS2 contribute to TMZ resistance through epigenetic regulation [194,195]. PSMG3-AS1 directly interacts with c-Myc, stabilizing it within the nucleus and promoting GB cell survival under TMZ treatment [196]. Similarly, lncRNAs and their interactions with miRNAs are critical in modulating chemotherapy drug sensitivity. We summarized the signal axis or miRNA regulated by lncRNA for TMZ resistance in Table 2.

#### 3.1.2. Overcoming the Blood–Brain Barrier

Due to the special location of the brain tumor, the primary challenge for drug accumulation and targeting in brain tumors is the blood–brain barrier (BBB). This barrier consists of tight junctions and adherens junctions between brain microvascular endothelial cells, forming a physical barrier and efflux pumps on endothelial cells that actively transport exogenous substances back into the systemic circulation, thereby creating a biochemical barrier. As a result, the prognosis for brain tumor diagnosis remains poor, partly due to the BBB hindering early detection and effective drug delivery. Despite the identification of numerous genetic mutations in gliomas and other central nervous system (CNS) tumors, drugs designed to target these mutations struggle to penetrate and accumulate within the tumor because of the dual barriers. Therefore, there is an urgent need for new therapeutic agents or drug delivery systems to improve patient prognosis [216,217].

This limitation is gradually being overcome through advanced technologies such as nanotechnology and the rational design of biomaterials [218]. The development of novel bifunctional dendrimer-based drug delivery systems (DDS), with modifications and loading on nano-carriers, can activate anti-tumor immunity, effectively cross the BBB, and inhibit GB [219]. MoS2-based nanocomposites demonstrate excellent control of drug release and can specifically respond to the tumor microenvironment. These materials have broad applications in tumor diagnosis and therapy, including in areas such as biosensors, bioimaging, chemotherapy, phototherapy, microwave hyperthermia, and combination therapies [220]. Intravenous treatment with cerium oxide nanoparticles coated with antisense oligonucleotides targeting lnc-HLX-2-7 can inhibit tumor growth. Combined with standard cisplatin therapy, it further suppresses tumor growth and significantly extends mouse survival [48].

Exosomes are extracellular vesicles (EVs) that contain proteins, nucleic acids, DNA, RNA, and other components derived from donor cells. The intrinsic properties of exosomes, which regulate complex intracellular pathways, enhance their potential utility in a variety of diseases. They are involved in immune responses, central nervous system-related disorders, and cancer progression [221,222,223,224,225]. Due to their low immunogenicity, low toxicity, intrinsic ability to cross biological barriers, and targeting capabilities, exosomes can be engineered to deliver various therapeutic payloads. Exosomes exhibit excellent pharmacokinetic properties and bioavailability while reducing the side effects of free drugs. Although challenges remain, exosomes hold significant promise for clinical application [226,227,228,229].

In glioblastoma, exosomes containing lncRNAs induce microglia to produce complement C5, promoting chemotherapy resistance [230]. Exosomes packaging the highly expressed LINC00473 transfer chemotherapy resistance to adjacent TMZ-sensitive GB cells. This reveals a new CREB/LINC00473/CEBPα/MGMT pathway. Furthermore, a chemotherapy resistance transmission mechanism based on exosomes has been uncovered, suggesting that LINC00473 could serve as a novel therapeutic target for GB [231]. Exosome-derived SBF2-AS1 acts as a ceRNA to lower the expression of miRNA-151a-3p, leading to upregulation of XRCC4 and subsequent TMZ resistance [232]. Additionally, the expression of Miat in stem-like medulloblastoma cells leads to resistance against treatment by downregulating the p53 pathway and diminishing the effectiveness of radiation-induced cell death. This can be reversed through therapeutic inhibition of Miat using antisense oligonucleotides [40].

### 3.2. Radiosensitivity

Radiotherapy is commonly used to treat various malignant tumors, but tumor cells show different levels of resistance to it, affecting patient prognosis. Cancer radiotherapy resistance directly impacts the efficacy of radiotherapy and is closely associated with poor patient prognosis. LncRNAs regulate radiotherapy responses by modulating key signaling pathways [233]. LINC01057, a modulator of NF-κB signaling, promotes mesenchymal differentiation in tumors. Knockdown of LINC01057 inhibits proliferation, invasion, and radioresistance in glioblastoma cells and suppresses tumor growth in vivo [234]. Through the miR-642a-5p/Notch1 axis, MAFG-AS1 contributes to enhancing radioresistance in GB cells [235].

Glioblastoma stem-like cells are critical for GB tumorigenesis and therapy resistance. Increasing evidence supports the oncogenic roles of lncRNAs. Through multi-omics analysis, loss of DARS1-AS1 inhibits GB cell/GSC proliferation and self-renewal while enhancing the radiosensitivity of GB cells/GSCs. The DARS1-AS1/YBX1 axis may serve as a therapeutic target to make GB sensitive to radiation/HR-deficiency-targeted therapies [236].

Similarly, overexpression of LINC00839 in GSCs is associated with GB progression and radioresistance. LINC00839 acts as a scaffold, promoting the c-Src-mediated phosphorylation of β-catenin, activating Wnt/β-catenin signaling. Combined treatment with the Wnt/β-catenin inhibitor celecoxib enhances the radiosensitivity of GSCs [237]. Under certain circumstances, GSCs can transition between mesenchymal and proneural subtypes. MES GSCs display greater malignancy and radioresistance and closely relate to an immunosuppressive microenvironment. MIR222HG interacts with the YWHAE/HDAC5 complex, facilitating the MES transition and radioresistance of GSCs through H4 deacetylation. Additionally, miR221 and miR222, co-transcribed with MIR222HG, can be delivered to macrophages via exosomes to target SOCS3, thereby leading to immunosuppressive polarization [238].

Furthermore, overexpression of RBPMS-AS1 inhibits tumor growth and Ki67-positive expression before and after radiotherapy [164]. Multi-dataset analysis has shown that HOTAIRM1 sensitizes GB cells to radiation [239] and identified potential targets and mechanisms for tumor-treating electric fields in GB [240].

### 3.3. Immunotherapy

In recent years, the application of monoclonal antibodies and immune checkpoint inhibitors has enhanced the immune system’s ability to target tumor cells. However, immune evasion remains a significant challenge in brain tumors. As crucial regulators within the tumor immune microenvironment, long non-coding RNAs hold substantial potential for improving immunotherapy in brain tumors.

The expression of NEAT1 has been associated with the response of GB patients to PD-1/PD-L1 therapy, underscoring the important role of lncRNAs in the tumor microenvironment [241]. PTRF enhances the stability and expression of NEAT1, inducing the NF-κB and PD-L1 signaling pathways that contribute to immune evasion [242]. Exosomes derived from glioma stem cells remodel glioma-associated macrophages through the NEAT1/miR-125a/STAT3 pathway [243]. Hypoxia-induced ALKBH5 upregulates CXCL8/IL8 expression via NEAT1, which enhances TAM recruitment and immune suppression, providing a potential immunotherapeutic strategy for treating GB [244]. Similarly, PVT1 regulates STAT1 and CX3CL1 to promote glioblastoma multiforme proliferation and shape an immune-suppressive tumor microenvironment (TME). Targeting PVT1 may offer promising therapeutic prospects for GB patients [245].

N6-methyladenosine (m6A) and lncRNAs play crucial roles in patients’ survival and their response to immunotherapy. A risk model based on m6A-related lncRNAs is associated with immune status, immune-suppressive biomarkers, and chemotherapy sensitivity in GB patients, making it a potential tool for clinical intervention [246]. m6A contributes to the rapid adaptation of the tumor microenvironment during cancer progression. Hypoxia-induced ALKBH5 removes m6A deposition on NEAT1, ultimately upregulating CXCL8/IL8 expression and inducing tumor immune microenvironment remodeling, providing a potential immunotherapeutic strategy for treating glioblastoma [244].

As previously mentioned, the function and application of lncRNAs are closely related. Long non-coding RNAs that “encode” micropeptides play a regulatory role in immunotherapy. H19 encodes an immune-related protein designated as H19-IRP. This protein facilitates immune suppression in GB by recruiting myeloid-derived suppressor cells (MDSCs) and tumor-associated macrophages (TAMs), resulting in T-cell exhaustion and the establishment of an immunosuppressive TME. A circular RNA vaccine targeting H19-IRP (circH19-vac) activates a strong cytotoxic T-cell response against GB and suppresses tumor growth [247].

## 4. Conclusions and Future Prospects

This review provides a comprehensive overview of the functional, prognostic, and therapeutic advancements associated with long non-coding RNAs (lncRNAs) in malignant brain tumors, emphasizing the potential role of the competitive endogenous RNA (ceRNA) network. LncRNAs exhibit diverse functionalities that facilitate their interaction with mRNA, proteins, and other molecular entities, thereby regulating several critical signaling pathways in tumor biology. They play pivotal roles in a variety of cellular processes, including tumor cell proliferation, migration, invasion, epithelial–mesenchymal transition (EMT), apoptosis, metabolism, immunity, and ferroptosis, all of which can either facilitate or inhibit tumor initiation and progression. Furthermore, lncRNAs are closely associated with patient survival and serve as essential biomarkers for diagnosis, prognosis, and therapeutic targeting. In the context of clinical treatment, lncRNAs have been shown to enhance the sensitivity of chemotherapy, particularly to TMZ, and they also contribute significantly to the efficacy of radiotherapy and immunotherapy.

The clinical application of lncRNA-based diagnostics and therapies holds immense promise. We discuss the potential of nanocarriers and exosomes in overcoming the blood–brain barrier (BBB) and enhancing drug efficacy. Exosomes show promise as effective clinical diagnostic tools and carriers for drug delivery. However, challenges remain in the efficient and cost-effective isolation and purification of sufficient quantities of exosomes, which hinder their clinical application. Therefore, it is essential to promote the use of standardized methods for isolating and purifying exosomes, such as efficient microfluidic devices [248,249,250].

The review further highlights research into multi-dimensional analysis, combining databases, multi-omics data, and clinical data to identify prognostic molecular biomarkers. In clinical applications, by combining high-throughput sequencing data with machine learning algorithms, it will be possible to establish robust diagnostic and prognostic tools that offer precision in patient stratification and treatment planning. Integrating imaging with multi-omics analysis and establishing efficient, self-learning predictive models—or developing high-sensitivity and specificity sensors—could significantly enhance the accuracy and convenience of diagnosis and prognosis [251,252,253].

Additionally, the potential of natural molecules and their derivatives in clinical therapy should not be overlooked. For example, flavonoids can prevent angiogenesis, inhibit cancer cell proliferation, and protect neuronal cells. They also exhibit the ability to regulate epigenetic modifications through lncRNAs, induce apoptosis and autophagy in GB cells, improve metabolic abnormalities in GB, interfere with the tumor microenvironment and related signaling pathways, and suppress angiogenesis in GB cells. Ultimately, these compounds may block tumor initiation, growth, proliferation, differentiation, invasion, and metastasis [254].

However, despite some advances, significant challenges remain. Many current studies rely on in vitro models or overexpression systems that may not accurately replicate physiological conditions. The scalability and reproducibility of lncRNA-targeted therapies and their long-term safety profiles require thorough investigation in clinical trials.

Due to the length and structure of the review, our discussion includes only a subset of lncRNA research and their interactions with miRNAs. There is no inclusion of emerging areas such as tRNAs and enhancer RNAs. Additionally, this review does not cover many studies on lncRNAs related to brain tumors. Several important lncRNAs or related protein families that warrant in-depth discussion have not been systematically covered, such as the SOX family [255], H19 [256], HOTAR [257], MALAT1 [258], and NEAT1 [259], among others, which have been widely studied.

In conclusion, lncRNAs represent a rapidly evolving field with profound implications for understanding and treating malignant brain tumors. As research progresses, the combination of innovative delivery systems, advanced computational tools, and integrative multi-omics approaches will be key to unlocking the full potential of lncRNA-based diagnostics and therapies, ultimately transforming the landscape of cancer treatment.

## Figures and Tables

**Table 1:** lncRNA-miRNA regulatory network in malignant brain tumors (LncRNAs marked with an asterisk (*) represent tumor-suppressing lncRNAs, while those without the asterisk represent tumor-promoting lncRNAs).

lncRNA	miRNA	Progression	Cancer
TP73-AS1	miR-494-3p	Promotes tumor progression [110]	Medulloblastoma
HOTAIR	miR-1/miR-206	Tumor growth, migration, invasion, and apoptosis [111]	Medulloblastoma
CRNDE	miR-29c-3p	Proliferation, migration, invasion, apoptosis, and chemosensitivity [112]	Medulloblastoma
IMAT1	hsa-miR22-3p	Invasiveness [113]	Meningioma
MALAT1	miR-145	Invasiveness [114]	Meningioma
MALAT1	miR-199a	Cell proliferation and apoptosis (associated with prognosis) [115]	GB
MEG3 *	miR-29c	Cell cycle, migration, invasion, and proliferation [116]	Meningioma
MEG3 *	miR-6088	Proliferation and EMT [117]	Glioma
SNHG1	miR-556-5p	Tumorigenesis [118]	Meningioma
SNHG1	miR-9-5p	Tumorigenesis and angiogenesis [119]	GB
SNHG4	miR-138	Proliferation [120]	GB
SNHG7	MicroRNA-449b-5p	Motility, migration, and invasion [121]	GB
NEAT1	miR-98-5p	Cancer progression [122]	GB
NEAT1	miR-128-3p	Occurrence and progression [123]	GB
HOXA-AS2	miR-885-5p	Tumorigenesis [124]	GB
HOXA-AS2	miR-2116-3p	Growth and proliferation [125]	GB
HOXA-AS3	miR-455-5p	Proliferation, migration, invasion, and prognosis [126]	GB
XIST	miR-448	Tumorigenesis [127]	GB
XIST	miR-126	Cell viability, migration, invasion, apoptosis resistance, and glucose metabolism [128]	GB
XIST	miR-133a	Proliferation and metastasis [129]	GB
XIST	miR-152	Proliferation and stemness [130]	GB
OXCT1-AS1	miR-195	Tumor progression [131]	GB
SEMA3B-AS1 *	miR-195	Tumor progression [132]	GB
LINC00460	miR-539	Proliferation, metastasis, and malignant transformation [133]	Malignant meningioma
LINC00702	miR-4652-3p	Tumor progression [134]	Malignant meningioma
LINC01426	miR-345-3p	Cell proliferation and prognosis [135]	GB
LINC00294 *	miR-1278	Cell proliferation [136]	Glioma
LINC00641 *	miR-4262	Cell proliferation and apoptosis [137]	Glioma
LINC00511	miR-524-5p	Cell proliferation, EMT, and prognosis [138]	GB
LINC01198	miR-129-5p	Cell proliferation and apoptosis [139]	GB
LINC01579	miR-139-5p	Cell proliferation and apoptosis [140]	GB
LINC00115	miR-200	Key regulators of self-renewal and tumorigenicity of GSCS [141]	GB
LINC00606	miR-486-3p	Proliferation, migration, and apoptosis [142]	GB
BCAR4	miR-2276	Invasion, tumorigenesis, and prognosis [143]	GB
CHRM3-AS2	miRNA-370-5P	Proliferation, invasion, and migration [144]	GB
LGMNP1	miR-495-3p	Proliferation and invasion [145]	GB
HOTAIRM1	miR-153-5p	Migration and invasion and potential molecular markers [146]	GB
LINC01393	miR-128-3p	Cell proliferation, migration, and invasion [147]	GB
Linc01094	miR-126-5p	Growth and invasion [148]	GB
DCST1-AS1	miR-29b	Cell proliferation [149]	GB
SATB2-AS1 *	miR-671-5p	Cell proliferation and apoptosis and glycolysis metabolism [150]	Low-grade glioma and GB
FEZF1-AS1	miR-363-3p	Cell proliferation and apoptosis and glycolysis metabolism [151]	GB
PSMA3-AS1	miR-411-3p	Proliferation and apoptosis [152]	GB
LPP-AS2	miR-7-5p	Tumorigenesis [153]	Glioma
HNF1A-AS1	miR-32-5p	Tumor progression [154]	Glioma
HAS2-AS1	miR-608	Migration, invasion, and prognosis [155]	GB
KTN1-AS1	miR-505-3p	Proliferation and invasion [156]	GB
HOXD-AS2	miR-3681-5p	Proliferation, migration, and invasion [157]	GB
HNF1A-AS1	miR-22-3p	Malignant behavior of tumors [158]	GB
DLGAP1-AS1	miRNA-515-5p	Tumor progression [159]	GB
OIP5-AS1	miR-495-3p	Angiogenesis [160]	GB
FLVCR1-AS1	miR-30b-3p	Proliferation, invasion, novel therapeutic targets, and diagnostic biomarkers [161]	GB
LEF1-AS1	miR-543	Colony formation, invasion, and migration [162]	GB
HAS2-AS1	miR-137	Cell proliferation and tumorigenicity [163]	GB
RBPMS-AS1 *	miR-301a-3p	Cell proliferation, apoptosis, and radiosensitivity [164]	GB
FEZF1-AS1	miR-34a	Invasion and migration [165]	GB
PSMB8-AS1	miR-22-3p	Cell proliferation, apoptosis, and radiation resistance [166]	GB
FGD5-AS1	miR-129-5p	Proliferation, migration, and EMT progression [167]	GB
GAS5 *	miR-let-7e and miR-125a	Cell migration and invasion, stemness and proliferation of GSCs, and prognostic biomarkers [168]	GB
MUF	miR-34a	Proliferation, migration, and invasion; TMZ sensitivity; therapeutic target [169]	GB
TMEM161B-AS1	hsa-miR-27a-3p	Proliferation, migration, invasion, and apoptosis; ferroptosis; TMZ sensitivity [170]	GB
NONHSAT079852.2	hsa-miR-10401-3p	Cell proliferation and invasion, neoplasm progression and recurrence, diagnostic biomarkers, and therapeutic targets [171]	GB
Unigene56159	miR-194-5p	Proliferation and invasion and potential therapeutic targets [172]	GB
RP1-86C_11.7_	hsa-miR-144-3p	Cell proliferation and intracellular iron levels [173]	GB
MYCNOS	miR-216b	Cell proliferation [174]	GB
PITPNA-AS1	miR-223-3p	Cell proliferation and apoptosis and potential biomarkers for diagnosis and treatment [175]	GB
COX10-AS1	miR-1-3p	Cell proliferation, migration, and invasion and tumorigenesis [176]	GB
RP3-439F8.1	miR-139-5p	Cell proliferation, colony formation, invasion, and cell cycle [177]	GB
MAFG-AS1	miR-34a	Cell proliferation [178]	GB
UNC5B-AS1 *	miR-24-3p	Cell proliferation [179]	GB
AGAP2-AS1	miR-486-3p	Growth and metastasis [180]	GB

**Table 2:** LncRNA governs TMZ resistance (LncRNAs marked with an asterisk (*) represent those negatively correlated with TMZ resistance, while those without the asterisk represent lncRNAs negatively correlated with TMZ sensitivity).

LncRNA	Signal Axis/miRNA
Linc00942	TPI1/SOX9 [197]
NEAT1	Connexin 43 [198]
CRNDE	LC3 II/I, Beclin-1, ATG-5, p62 [199]
XLOC013218	PI3K/AKT [200]
ZBED3-AS1 *	THBD [201]
LINC00520	RNA-binding protein LIN28B [202]
PVT1	JAK/STAT [203]
MAGI2-AS3 *	Akt pathway [204]
LINC00174	miR-138-5p/SOX9 [205]
HOTAIR	miR-526b-3p [206]
HOTAIR	mir-125 [207]
MIR99AHG	miR-204-5p [208]
TUSC7 *	miR-10a [209]
LINC01410	miR-370-3p [210]
UCA1	miR-182-5p [211]
LINC00511	miR-126-5p [212]
OIP5-AS1	miRNA-129-5p [213]
SNHG12	miR-129-5p [214]
HOXA-AS2	miR-302a-3p/IGF1 [215]
